# Indocyanine Green Fluorescence-Guided Laparoscopic Subtotal Gastrectomy for Remnant Gastric Cancer after Pancreaticoduodenectomy: A Case Report

**DOI:** 10.70352/scrj.cr.26-0512

**Published:** 2026-10-20

**Authors:** Osamu Inamoto, Shuhei Sakamoto, Takashi Fujimoto, Kazuhiro Nishiyama, Ryotaro Ogawa, Atsushi Yoshizawa, Yoshiro Taki, Izumi Komoto

**Affiliations:** Department of Surgery, Kansai Electric Power Hospital, Osaka, Osaka, Japan

**Keywords:** remnant gastric cancer, pancreaticoduodenectomy, indocyanine green, laparoscopic subtotal gastrectomy, fluorescence-guided surgery

## Abstract

**INTRODUCTION:**

Remnant gastric cancer (RGC) is rare after pancreaticoduodenectomy (PD). Surgical management of such cases is technically challenging because of postoperative adhesions and altered anatomy due to reconstruction. Although a completion total gastrectomy is the standard treatment for RGC, it can be highly invasive, particularly in elderly patients with significant comorbidities. While function-preserving subtotal gastrectomy or minimally invasive approaches have been considered, they remain technically demanding. Recent advances in indocyanine green (ICG) fluorescence imaging have facilitated precise tumor localization and real-time tissue perfusion assessment in complex surgical settings.

**CASE PRESENTATION:**

A 75-year-old man with a 24-year survival history after PD for pancreatic cancer presented with early-stage RGC (0–IIa, size 35 mm, invading the submucosa). The patient was at high risk, with a history of cerebral infarction requiring dual antiplatelet therapy and long-term corticosteroid therapy for pemphigus vulgaris. A laparoscopic subtotal gastrectomy was performed. Preoperative endoscopic ICG fluorescent clipping guided the determination of an adequate surgical margin under near-infrared visualization, despite dense adhesions. ICG fluorescence enabled precise tumor localization and confirmation of adequate perfusion, thereby facilitating stomach-preserving resection. Although the postoperative course was complicated by a Grade B pancreatic fistula that was managed conservatively, the patient was discharged.

**CONCLUSIONS:**

Laparoscopic subtotal gastrectomy with ICG fluorescence guidance can be considered a technically useful surgical option for RGC, even 24 years after PD. This technique provides precise tumor localization and real-time perfusion assessment, assisting surgical resection in high-risk patients with multiple comorbidities and complex reoperative anatomy.

## Abbreviations

CFS
: clinical frailty scale
DAPT
: dual antiplatelet therapy
ECOG PS
: Eastern Cooperative Oncology Group Performance Status
ICG
: indocyanine green
ISGPS
: International Study Group of Pancreatic Surgery
LGA
: left gastric artery
MIS
: minimally invasive surgery
NIR
: near-infrared
PD
: pancreaticoduodenectomy
PDAC
: pancreatic ductal adenocarcinoma
POPF
: postoperative pancreatic fistula
RGC
: remnant gastric cancer
UICC
: Union for International Cancer Control

## INTRODUCTION

With recent improvements in long-term survival after upper gastrointestinal surgery, the incidence of RGC has steadily increased.^1,2)^ However, RGC occurring after PD remains extremely rare.^3,4)^

While completion total gastrectomy has historically been the standard treatment for RGC, MIS and function-preserving subtotal gastrectomy have increasingly gained attention to mitigate surgical invasiveness and preserve postoperative QOL.^5–7)^ Nevertheless, performing laparoscopic subtotal gastrectomy after PD remains technically challenging because of dense adhesions, complex anatomical alterations around the infrapancreatic area, and prior reconstruction routes that increase the risk of inadvertent vascular or organ injury.

We report herein a case of RGC that occurred 24 years after PD in a patient with multiple comorbidities who underwent laparoscopic subtotal gastrectomy using multimodal ICG fluorescence navigation.

## CASE PRESENTATION

A 75-year-old man had a history of PD performed in our department for PDAC and had been undergoing regular follow-up. He was asymptomatic, but gastric cancer on the oral side of the gastrojejunostomy was detected incidentally during routine screening endoscopy. Endoscopic findings revealed a 0–IIa lesion measuring 35 mm with suspected invasion of the submucosa. Endoscopic treatment was not indicated because of the tumor depth, and surgical resection was planned.

The patient had a complex medical history featuring severe physical frailty secondary to an old cerebral infarction, which resulted in chronic neurological deficits, an ECOG PS^8)^ of 3, and a CFS score^9)^ of 6 (moderately frail). Furthermore, he had been receiving long-term oral corticosteroid therapy (prednisolone, 4 mg/day) for more than 20 years to manage pemphigus vulgaris and was on DAPT for cerebrovascular disease.

His past medical history included colorectal cancer at age 35 and pancreatic cancer at age 51. Although multiple primary malignancies were diagnosed, a hereditary cancer syndrome (such as Lynch syndrome) was considered unlikely given the absence of a relevant family history.

His surgical history was highly complex and involved multiple stages. 40 years before the current admission, he underwent sigmoid colectomy for sigmoid colon cancer, combined with ileocecal resection and cholecystectomy. Furthermore, 24 years before the current admission, the patient underwent open PD combined with en bloc resection of the ileocolic anastomosis and subsequent reanastomosis of the colon due to tumor invasion. To ensure precise intraoperative tumor localization within severe adhesions, ICG-labeled clips were endoscopically placed adjacent to the proximal side of the tumor before surgery (**Fig. 1**). Contrast-enhanced CT revealed complex anatomical alterations resulting from the previous PD, including gastrojejunostomy and pancreaticojejunostomy, with no evidence of regional lymphadenopathy or distant metastasis (**Fig. 2**). With a diagnosis of early RGC, laparoscopic surgery was scheduled. Perioperative heparin bridging was performed to minimize thromboembolic and hemorrhagic risks.

**Fig. 1:**
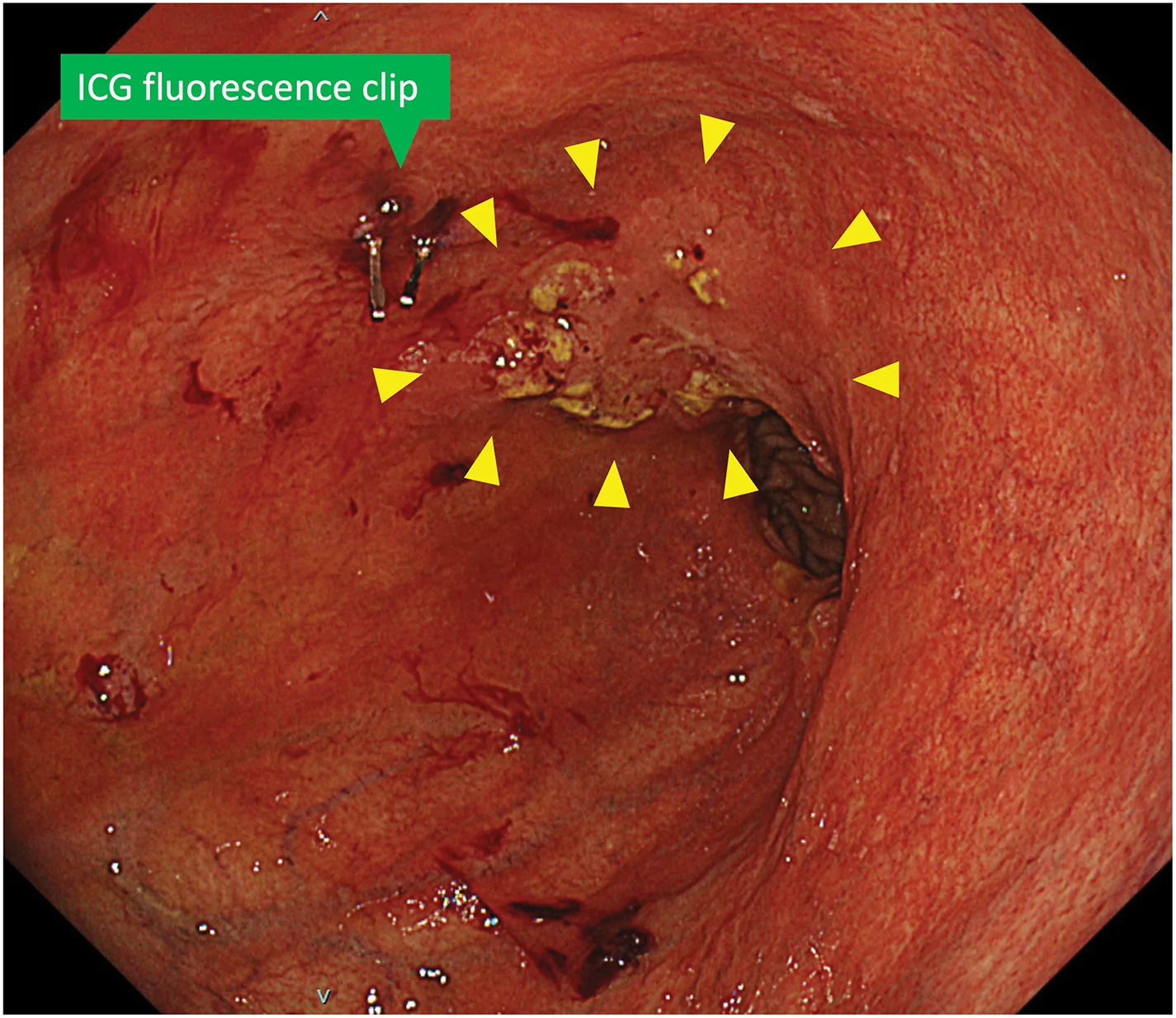
Preoperative upper gastrointestinal endoscopy showing early RGC (yellow arrowheads). Endoscopically placed ICG-labeled clips are visible near the previous gastrojejunostomy. ICG, indocyanine green; RGC, remnant gastric cancer

**Fig. 2:**
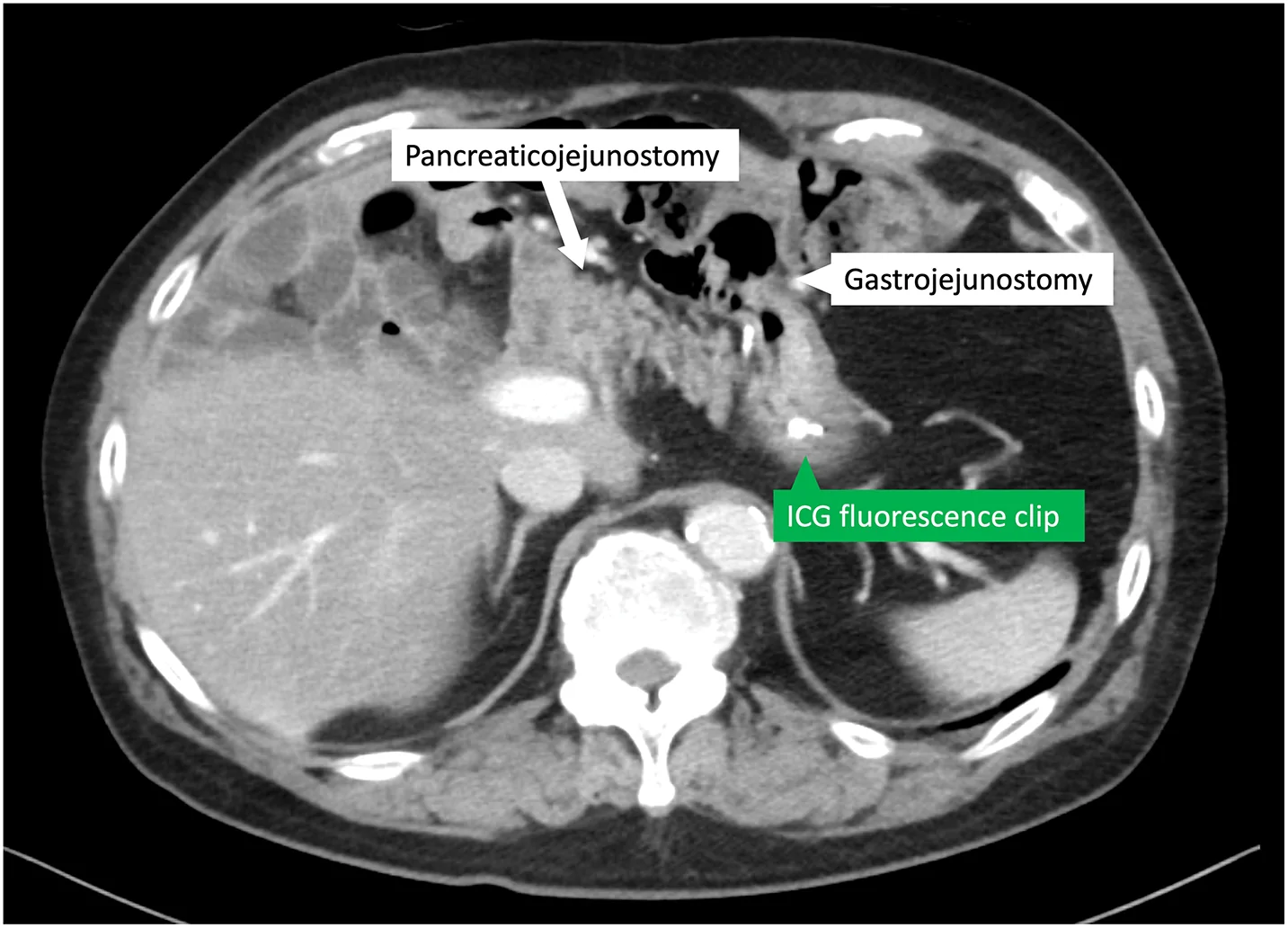
Contrast-enhanced abdominal CT demonstrating the postoperative anatomy after PD. The previous gastrojejunostomy and pancreaticojejunostomy sites are indicated by arrowheads. PD, pancreaticoduodenectomy

### Surgical procedure

Under general anesthesia, an initial open laparotomy with a small incision was performed in the suprapubic region to establish pneumoperitoneum. Initial laparoscopic inspection confirmed extensive dense adhesions throughout the abdomen. A working port was added to the left lower quadrant to perform adhesiolysis.

As extensive adhesions were carefully dissected and the intra-abdominal workspace was gradually expanded, additional ports were sequentially introduced under direct laparoscopic vision to optimize surgical triangulation for gastrectomy (**Fig. 3A**).

**Fig. 3:**
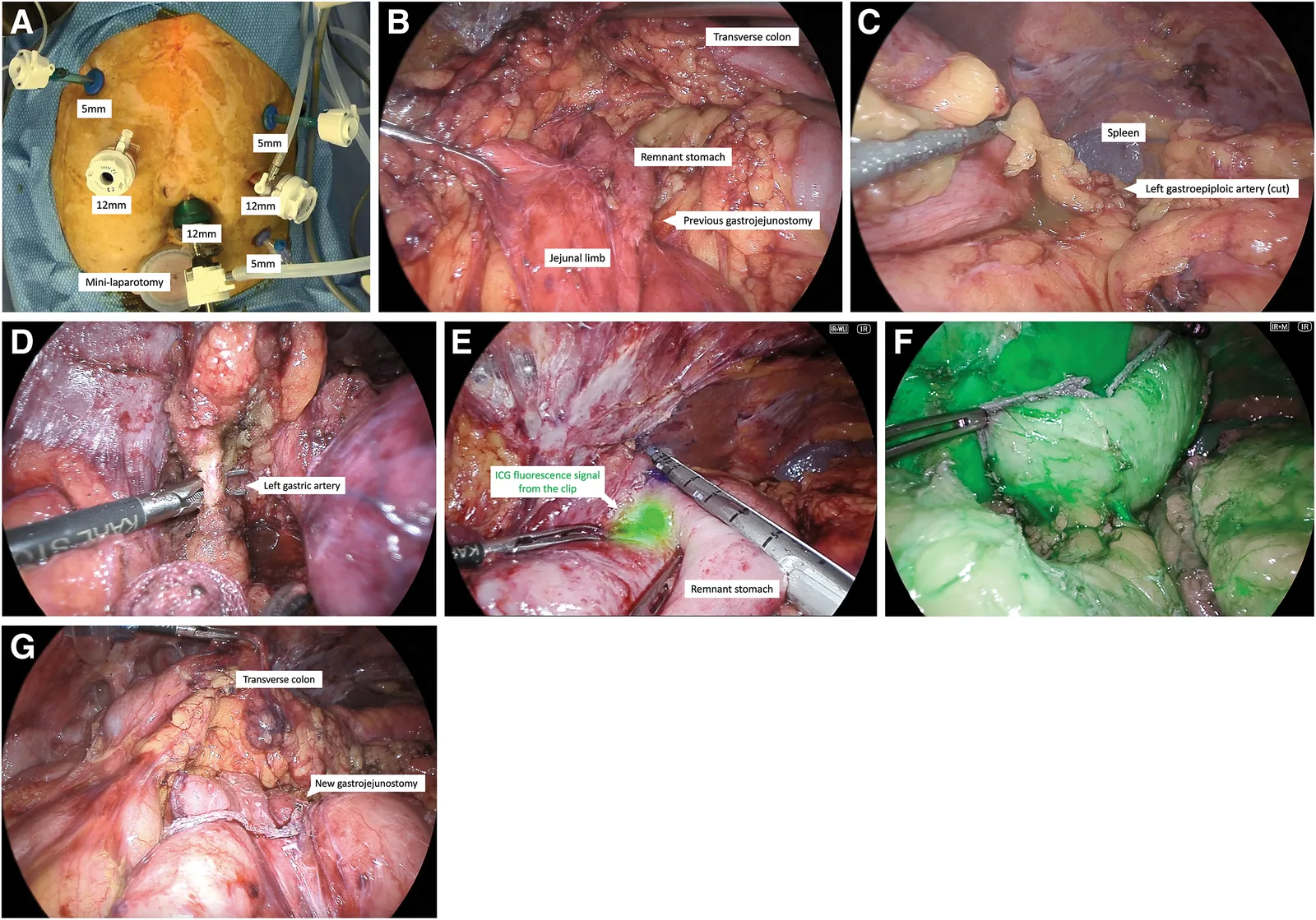
Intraoperative laparoscopic findings and surgical procedures. (**A**) Port configuration, including a camera port at the umbilicus, the E·Z ACCESS (Hakko Co, Ltd., Tokyo, Japan) system at the suprapubic mini-laparotomy site, and 5 working ports. (**B**) Dissection of dense adhesions among the remnant stomach, transverse colon, previous gastrojejunostomy, and jejunal limb. (**C**) Lymph node dissection along the LGEA (station no. 4sb). (**D**) Lymph node dissection along the LGA (station no. 7). (**E**) NIR ICG fluorescence imaging showing the signal from the endoscopically placed clip through the gastric wall, facilitating determination of the stapler line. (**F**) Real-time ICG fluorescence angiography confirming adequate perfusion of the proximal gastric stump. (**G**) Reconstruction by functional end-to-end gastrojejunostomy using the retrocolic Roux-en-Y method. ICG, indocyanine green; LGA, left gastric artery; LGEA, Left gastroepiploic artery; NIR, near-infrared

Dense adhesions between the liver, transverse colon, remnant stomach, and jejunal limbs were carefully dissected. The previous gastrojejunostomy was identified as a retrocolic reconstruction (**Fig. 3B**). After mobilizing the gastrojejunostomy, the afferent and efferent limbs were divided using a linear stapler. The gastrojejunostomy complex was pulled cranially through the transverse mesocolic defect. The previous pancreaticojejunostomy and choledochojejunostomy were not visualized during the procedure.

Lymphadenectomy was performed at stations 1, 3, 4sb, and 7. The left gastroepiploic vessels (**Fig. 3C**) and LGA (**Fig. 3D**) were divided at their origins, despite dense postoperative scarring.

The laparoscopic system was switched to NIR imaging to determine the appropriate proximal transection line with an adequate oncological margin. The ICG fluorescence signal emitted from the endoscopically preplaced clips was clearly visualized through the gastric wall as a localized bright green area. Guided precisely by this fluorescent navigation, a laparoscopic linear stapler was positioned oral to the ICG signal, and the remnant stomach was transected (**Fig. 3E**).

Following gastric transection and specimen removal, an ICG bolus was injected intravenously to evaluate the microvascular blood supply to the residual gastric stump. NIR visualization confirmed rapid and excellent capillary tissue perfusion across the remaining proximal stomach up to the fresh staple line (**Fig. 3F**).

Reconstruction was performed using the laparoscopic Roux-en-Y method. Due to limited jejunal mobility, the existing retrocolic route was reused to avoid anastomotic tension (**Fig. 3G**). The overall surgical schema illustrating the preoperative and postoperative anatomical relationships is presented in **Fig. 4**.

**Fig. 4:**
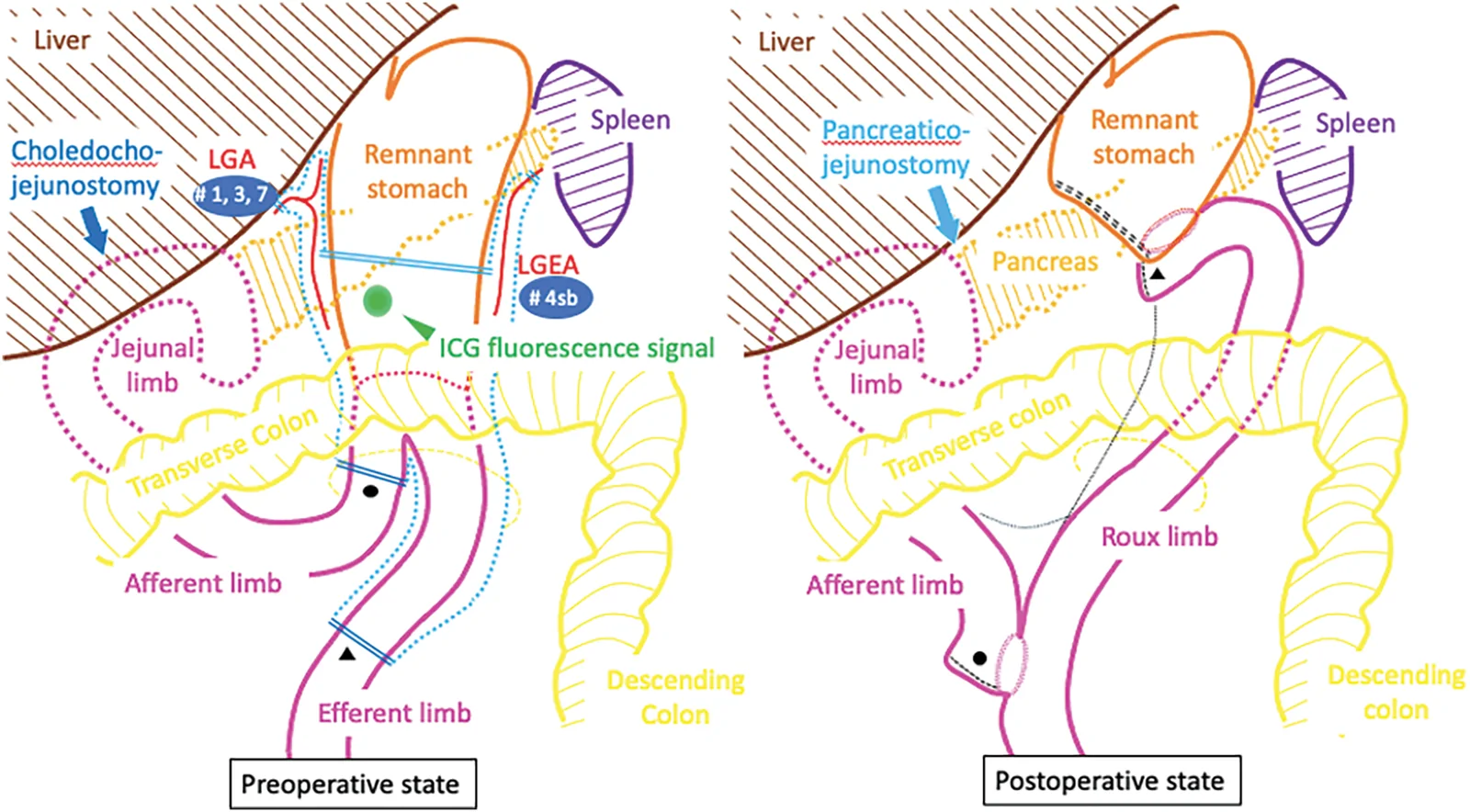
Schematic illustration of the surgical strategy for RGC after PD. (Left) Preoperative anatomy showing the previous reconstructive routes (choledochojejunostomy, pancreaticojejunostomy, and retrocolic gastrojejunostomy), tumor location, and planned lymph node dissection areas (stations 1, 3, 4sb, and 7). (Right) Postoperative anatomy after laparoscopic subtotal gastrectomy with retrocolic Roux-en-Y reconstruction, performed without exposing the pancreaticojejunostomy or choledochojejunostomy. ICG, indocyanine green; LGA, left gastric artery; LGEA, Left gastroepiploic artery; PD, pancreaticoduodenectomy; RGC, remnant gastric cancer

Finally, the resected specimen was placed in an extraction bag and retrieved through the initial small laparotomy site in the suprapubic region to avoid additional upper abdominal incisions. The total operative time was 5 h 59 min, with an estimated blood loss of 100 mL. No intraoperative complications were observed.

### Pathological findings and postoperative course

Gross examination of the resected specimen revealed a well-demarcated lesion located near the gastrojejunostomy site, with endoscopically placed ICG-labeled clips firmly attached to the adjacent mucosa (**Fig. 5**). Histopathological evaluation confirmed well- to moderately differentiated tubular adenocarcinoma. The tumor invaded the muscularis propria (pT2), with negative surgical margins (R0 resection) and no regional lymph node metastasis (pN0). According to the 8th edition of the UICC TNM staging system,^10)^ the final pathological stage was Stage IB (pT2N0M0).

**Fig. 5:**
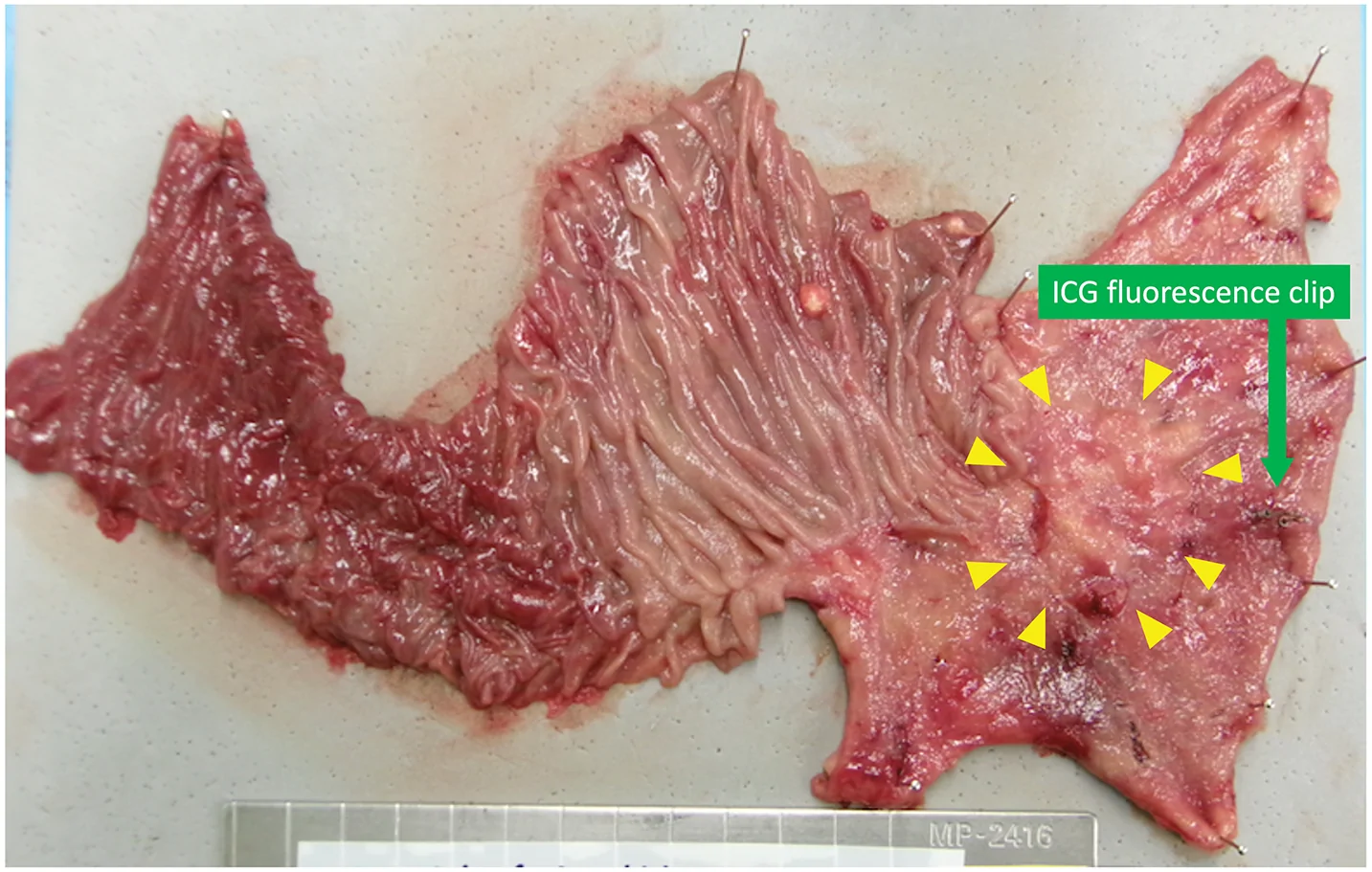
Macroscopic view of the resected specimen showing the gastric cancer lesion (yellow arrowheads) and endoscopically placed ICG-labeled clips. ICG, indocyanine green

The postoperative course was notable for multiple adverse events that were systematically managed. The patient developed a Grade B POPF according to the ISGPS criteria,^11)^ delayed jejunal limb transit, and aspiration pneumonia.

Regarding the POPF, the peak drain amylase level was 133270 U/L on POD 9. Contrast-enhanced CT revealed fluid collection around the superior border of the pancreas with surrounding inflammatory changes, likely resulting from extensive adhesiolysis around the suprapancreatic regions. The drain was removed on POD 40. Although pancreatic resection was not performed in the present procedure, the clinical presentation met the criteria for a clinically relevant pancreatic fistula requiring prolonged drain management and conservative therapy beyond normal postoperative care. Therefore, in accordance with the ISGPS definitions, as applied to this upper abdominal reoperation involving the pancreatic region, this complication was classified as a Grade B POPF.

These complications were managed conservatively and classified as Grade II according to the Clavien–Dindo classification.^12)^ Oral intake was ultimately restored, and the patient was transferred to a rehabilitation hospital on POD 92 without requiring reoperation or interventional radiology. During the 6-month postoperative follow-up period, he has maintained a good nutritional status with satisfactory oral intake, showing only minimal weight loss. There has been no clinical evidence of recurrence or late complications, such as adhesive bowel obstruction.

## DISCUSSION

Managing RGC following PD is difficult because of altered anatomy and severe adhesions. The primary novelty of this case report lies in demonstrating that, even under such a hostile surgical environment, minimally invasive and function-preserving gastrectomy can be successfully achieved with the support of NIR ICG fluorescence navigation.

With recent improvements in the long-term survival of patients undergoing upper gastrointestinal surgery, the clinical incidence and detection of secondary RGC have steadily increased.^1,2)^ Historically, completion total gastrectomy via an open approach has been considered the standard radical treatment; however, open reoperation is associated with significant morbidity and mortality due to severe abdominal scarring.^13)^

To mitigate surgical invasiveness while optimizing patient outcomes, surgical strategies have increasingly focused on function-preserving approaches. Recent evidence, such as the multicenter study by Kudou et al., highlights the validity and clinical benefits of subtotal gastrectomy and stomach-preserving strategies for RGC in achieving favorable short- and long-term outcomes while mitigating adverse nutritional impacts, such as excessive weight loss.^14)^

Minimally invasive gastrectomy has increasingly been performed to mitigate surgical invasiveness based on early foundational reports.^5)^ However, while recent national data indicate that MIS for RGC reduces intraoperative blood loss, it is also associated with technical challenges, such as longer operative times and a higher incidence of anastomotic leakage.^15)^

Furthermore, performing laparoscopic gastrectomy for RGC following PD introduces exceptional anatomical challenges. Beyond the dense adhesions typical of secondary RGC, prior PD results in severe scarring and altered pathways around the infra-pancreatic and hilar regions. These conditions markedly increase the risk of vascular or organ injury, rendering lymphadenectomy and mobilization difficult.

To our knowledge, only a few cases of minimally invasive gastrectomy following PD have been reported^16–19)^ (**Table 1**). Kim et al. described the first laparoscopic total gastrectomy for RGC after PD.^16)^ Subsequently, Shirakawa et al. reported laparoscopic gastrectomy for gastric mixed neuroendocrine–non-neuroendocrine neoplasm (MiNEN) after PD,^17)^ followed by Ito et al., who reported the first robotic gastrectomy for RGC after PD.^18)^ Furthermore, our group recently described a laparoscopic distal gastrectomy for gastric cancer following pylorus-preserving PD.^19)^ As summarized in **Table 1**, while minimally invasive approaches for RGC following PD are exceptionally demanding because of severe adhesions around previous anastomoses and require meticulous postoperative management, they can be safely accomplished by experienced surgical teams.

**Table 1:** Summary of published cases of minimally invasive gastrectomy for RGC following PD

Case	Author	Year	Age	Sex	Primary Disease	Interval (year)	PD		Gastrectomy			Complications	Outcome
							Type	Reconstruction	Approach	Extent of Resection	Reconstruction		
1	Kim^16)^	2016	73	M	Bile duct cancer	25	PD	PJ	L	TG	R-Y	–	7 days
2	Shirakawa^17)^	2022	63	M	P-NET	5	SSPPD	PJ	L	STG	R-Y	–	31 days
3	Ito^18)^	2023	89	M	IPMN	12	SSPPD	PJ	R	STG	R-Y	–	N/A
4	Inamoto^19)^	2026	85	F	Duodenal papillary cancer	14	PPPD	PJ	L	DG	R-Y	–	13 days
Present Case	Inamoto	2026	75	M	PDAC	24	PD	PJ	L	STG	R-Y	POPF, jejunal limb transit, aspiration pneumonia	92 days

DG, distal gastrectomy; F, female; IPMN, intraductal papillary mucinous neoplasm; L, laparoscopic; M, male; N/A, not applicable; P-NET, pancreatic neuroendocrine tumor; PD, pancreaticoduodenectomy; PDAC, pancreatic ductal adenocarcinoma; PJ, pancreaticojejunostomy; POPF, postoperative pancreatic fistula; PPPD, pylorus-preserving pancreaticoduodenectomy; R, robotic; RGC, remnant gastric cancer; R-Y, Roux-en-Y; SSPPD, subtotal stomach-preserving pancreaticoduodenectomy; STG, subtotal gastrectomy; TG, total gastrectomy

The optimal extent of gastric resection and lymph node dissection for RGC remains a subject of ongoing debate. While recent large-scale registry data highlight the importance of extended nodal clearance—including perigastric, suprapancreatic, and splenic hilar lymph nodes (station 10)—for advanced RGC,^20)^ no established evidence specifically defines the optimal dissection boundaries for RGC following PD.

In this case, we selected laparoscopic subtotal gastrectomy with selective dissection (stations 1, 3, 4sb, 4d, and 7). Because the tumor appeared clinically as an early-stage lesion (although it was finally diagnosed as pT2) in the middle gastric body without greater curvature invasion, stations 2, 4sa, and 10 were omitted. Subtotal gastrectomy with selective lymph node dissection was the most pragmatic approach to balance oncological curability with the avoidance of excessive surgical invasiveness in this high-risk reoperative setting after PD.

The implementation of this less invasive approach was facilitated by the multimodal application of ICG fluorescence imaging. Preoperative submucosal ICG marking has been shown to facilitate accurate tumor localization and determination of an appropriate resection margin during laparoscopic gastrectomy.^21)^ However, submucosal ICG tattooing is associated with rapid diffusion and limited longevity.^22)^ To overcome these limitations, fluorescent clip marking has been investigated as a durable alternative, and its feasibility for intraoperative tumor localization and determination of the resection line has been demonstrated.^23)^

This specific advantage of fluorescent clipping was particularly evident in the reoperative field after PD, where the serosa of the remnant stomach was covered by dense fibrous adhesions, making conventional tactile or visual identification of the tumor location from the laparoscopic field extremely difficult. Unlike free liquid dye, which may diffuse into surrounding tissues, the fluorescent signal from the clips remained localized and could be detected through the gastric wall under NIR light, allowing precise determination of the transection line.

Complementing this precise anatomical localization, the second key component of this multimodal ICG application was real-time intraoperative perfusion assessment. Adequate blood supply to the residual proximal gastric stump is essential for safe reconstruction and reduction of the risk of anastomotic complications. By combining fluorescent clip localization with real-time ICG fluorescence angiography, we were able to confirm satisfactory perfusion of the gastric stump before completing the reconstruction. This multimodal approach therefore addressed 2 critical challenges in this hostile reoperative field: accurate tumor localization and reliable assessment of gastric perfusion.

Although the patient experienced a prolonged postoperative course involving a Grade B POPF, delayed transit, and aspiration pneumonia, requiring 92 days until discharge because of pre-existing frailty, no reoperation or interventional procedures were necessary. Given the dense adhesions and complex reoperative anatomy around the previous PD site, attempting a completion total gastrectomy would have carried a substantial risk of extensive tissue injury and severe morbidity. Therefore, laparoscopic subtotal gastrectomy served as a technically feasible stomach-preserving option that avoided excessive physiological burden.

## CONCLUSIONS

Laparoscopic subtotal gastrectomy may be a feasible treatment option for select patients with RGC after PD. ICG fluorescence imaging is useful for intraoperative tumor localization and perfusion assessment, facilitating resection in the setting of complex postoperative anatomy.

## DECLARATIONS

### Availability of data and materials

The datasets supporting the conclusions of this article are included within the article and its additional files.
